# Targeted test evaluation: a framework for designing diagnostic accuracy studies with clear study hypotheses

**DOI:** 10.1186/s41512-019-0069-2

**Published:** 2019-12-19

**Authors:** Daniël A. Korevaar, Gowri Gopalakrishna, Jérémie F. Cohen, Patrick M. Bossuyt

**Affiliations:** 1 Department of Respiratory Medicine, Academic Medical Center, Amsterdam University Medical Centers, Amsterdam, the Netherlands; 2 Department of Epidemiology and Biostatistics, Vrije University Medical Centre, Amsterdam University Medical Centers, Amsterdam, the Netherlands; 3 Department of General Pediatrics and Pediatric Infectious Diseases, Necker-Enfants Malades Hospital, APHP, Paris Descartes University, Paris, France; 4 Inserm U1153, Obstetrical, Perinatal and Pediatric Epidemiology Research Team, Centre of Research in Epidemiology and Statistics Sorbonne Paris Cité (CRESS), Paris Descartes University, Paris, France; 5 Department of Clinical Epidemiology, Biostatistics and Bioinformatics, Academic Medical Center, Amsterdam University Medical Centers, Amsterdam, the Netherlands

## Abstract

Most randomized controlled trials evaluating medical interventions have a pre-specified hypothesis, which is statistically tested against the null hypothesis of no effect. In diagnostic accuracy studies, study hypotheses are rarely pre-defined and sample size calculations are usually not performed, which may jeopardize scientific rigor and can lead to over-interpretation or “spin” of study findings. In this paper, we propose a strategy for defining meaningful hypotheses in diagnostic accuracy studies. Based on the role of the index test in the clinical pathway and the downstream consequences of test results, the consequences of test misclassifications can be weighed, to arrive at minimally acceptable criteria for pre-defined test performance: levels of sensitivity and specificity that would justify the test’s intended use. Minimally acceptable criteria for test performance should form the basis for hypothesis formulation and sample size calculations in diagnostic accuracy studies.

## Introduction

The randomized controlled trial (RCT) has become the undisputed cornerstone of evidence-based health care [1]. RCTs typically evaluate the benefits and harms of pharmaceuticals (and other interventions) by comparing health outcomes between one group of participants who receive the drug to be evaluated, and a second group of participants who receive a placebo or an alternative drug [2]. Most RCTs have as a pre-specified hypothesis that the intervention under evaluation improves health outcomes, which is statistically tested against the null hypothesis of no effect (Table 1). The sample size of the trial is then calculated based on this pre-specified hypothesis and on the desired magnitude of the type I and type II errors [3]. Based on the collected data, investigators then typically calculate a test statistic and the corresponding *p* value. This is done alongside estimating effect sizes, such as the mean difference, relative risk, or odds ratio, and their precision, such as confidence intervals.

**Table 1:** Commonly used terminology in statistics of randomized controlled trials

Term	Explanation
Null hypothesis	Claims that there is no difference in outcome across two or more groups (e.g., drug A is as good as placebo)
Alternative hypothesis	Claims that there is a difference in outcome across two or more groups (e.g., drug A is better than placebo)
Type 1 error (α)	Rejection of a true null hypothesis (i.e., a false-positive result)
Type 2 error (β)	Failure to reject a false null hypothesis (i.e., a false-negative result)
Effect size	A quantitative measure of the magnitude of the effect (e.g., mean difference, relative risk, or odds ratio)
*p* value	Probability of obtaining the identified result (or something more extreme) under the assumption that the null hypothesis is true

The situation is very different for diagnostic tests. Comparative trials that focus on the effects of testing on patient outcomes are relatively rare [4]. There is, in general, no requirement to demonstrate a reasonable benefits-to-harms balance for new tests before they can be introduced to the market [5]. The clinical performance of medical tests is often evaluated in diagnostic accuracy studies. Such studies evaluate a diagnostic test’s ability to correctly distinguish between patients with and without a target condition, by comparing the results of the test against the results of a reference standard (Table 2) [6].

**Table 2:** Diagnostic accuracy studies

**Fig. 1:**
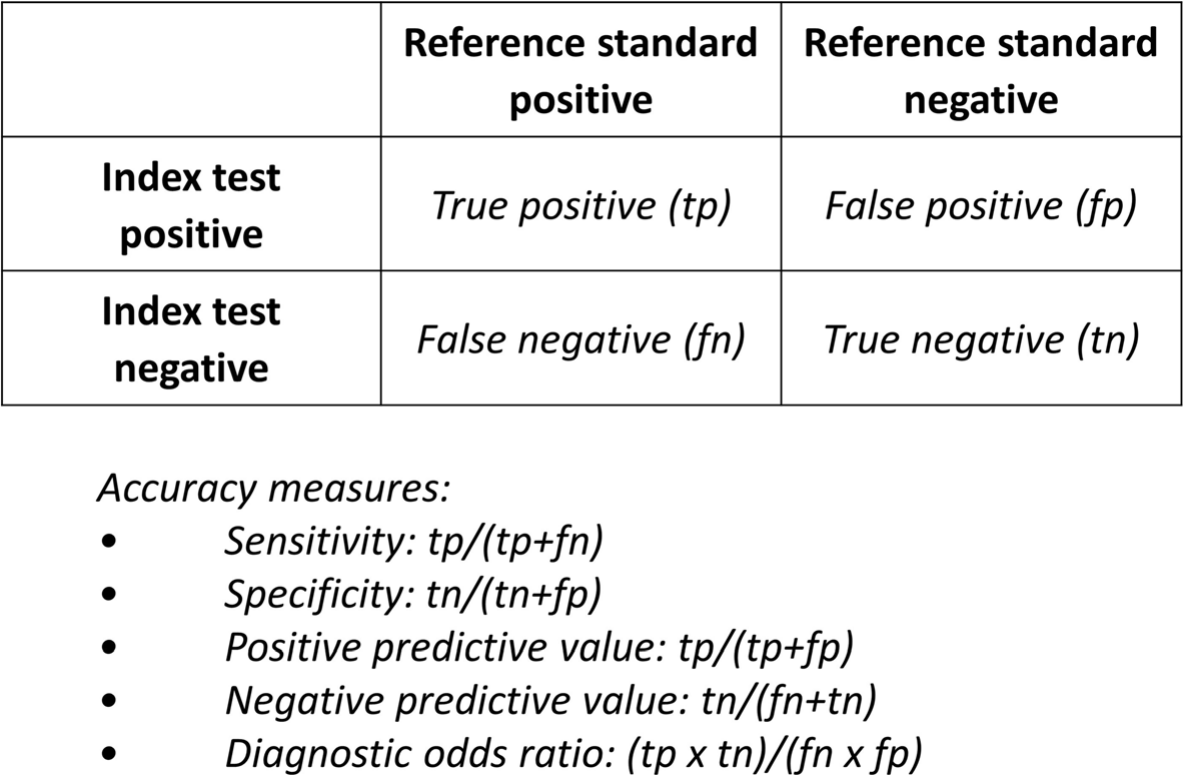
Typical output of a diagnostic accuracy study: the contingency table (or “2 × 2 table”)

**Fig. 2:**
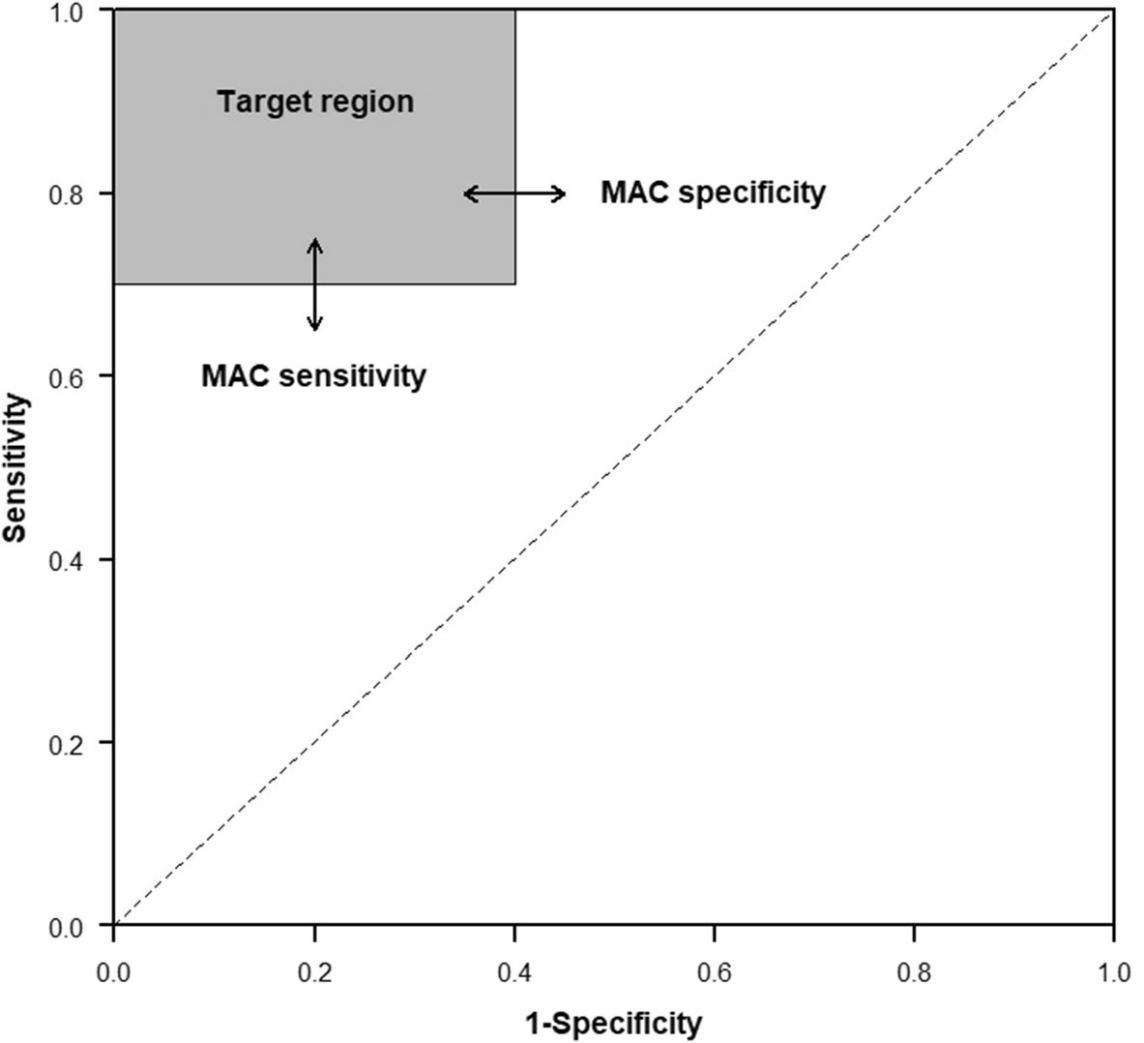
Receiver operating characteristic (ROC) space with “target region” based on minimally acceptable criteria for accuracy. ROC space has two dimensions: sensitivity (*y*-axis) and 1-specificity (*x*-axis). When the sum of sensitivity and specificity is ≥ 1.0, the test’s accuracy will be a point somewhere in the upper left triangle. The “target region” of a diagnostic accuracy study will always touch the upper left corner of ROC space, which is the point for perfect tests, where both sensitivity and specificity are 1.0. From there, the rectangle extends down, to MAC for sensitivity, and extend to the right, towards MAC for specificity. The gray square represents the target region of a diagnostic accuracy study with a MAC (sensitivity) of 0.70, and a MAC (specificity) of 0.60. MAC, minimally acceptable criteria

In diagnostic accuracy studies, a series of patients suspected of having a target condition undergo both an index test (i.e., the test that is being evaluated) and the clinical reference standard (i.e., the best available method for establishing if a patient does or does not have the target condition) [6]. Assuming that the results of the index test and reference standard are dichotomous—either positive or negative—we can present the results of the study in a contingency table (or “2 × 2 table”), which shows the extent to which both tests agree (Fig. 1). Discrepancies between the results of the index test and the reference standard are considered to be false-positive and false-negative index test results. Although it is possible to generate a single estimate of the index test’s accuracy, such as the diagnostic odds ratio [7], it is usually more meaningful to report two statistics: one for patients with the target condition, or sensitivity, and one for patients without the target condition, or specificity (Fig. 1). One reason is that the clinical consequences of misclassifications from false-positive and false-negative test results usually differ. As a visual aid, we can picture a test’s sensitivity and specificity as a point in the receiver operating characteristic (ROC) space, which has these two dimensions: sensitivity (*y*-axis) and 1-specificity (*x*-axis) (Fig. 2).	

Diagnostic accuracy studies typically report results in terms of accuracy statistics, such as sensitivity and specificity. Many fail to report measures of statistical precision [8]. Somewhat surprisingly, most diagnostic accuracy studies do not pre-specify a study hypothesis; they are usually reported without any explicit statistical test of a null hypothesis. In an analysis of 126 published diagnostic accuracy studies, Ochodo and colleagues observed that only 12% reported any statistical test of a hypothesis somewhat related to the study objectives, and no more than 11% reported a sample size justification [9]. Similar evaluations found that only 5% of diagnostic accuracy studies published in eight leading medical journals reported a sample size justification, and 3% of diagnostic accuracy studies of depression screening tools, and 3% of diagnostic accuracy studies in ophthalmology [10–12].

We believe the logic of having clear and pre-specified study hypotheses could and should extend to diagnostic accuracy studies. Scientific rigor is likely to benefit from this, as explicitly defining study hypotheses forces researchers to express minimally acceptable criteria for accuracy values that would make a test clinically fit-for-purpose, before initiating a study. A clearly defined study hypothesis also enables an informed judgment of the appropriateness of the study’s design, sample size, statistical analyses, and conclusions. It may also prevent the authors from over-interpreting their findings [9, 13, 14], as the absence of a pre-specified hypothesis leaves ample room for “spin”: generous presentations of the study findings, inviting the readers to conclude that the test is useful, even though the estimates of sensitivity and specificity do not support such a conclusion.

Below, we propose a strategy for defining meaningful hypotheses in diagnostic accuracy studies, based on the consequences of using the test in clinical practice. With the exposition below, we invite researchers who are designing diagnostic accuracy studies to derive meaningful study hypotheses and minimally acceptable criteria for test accuracy: targeted test evaluation.

## Meaningful hypotheses about diagnostic accuracy

Since there are typically two measures of accuracy in a diagnostic accuracy study (Table 2 and Fig. 1), we need a joint hypothesis, with one component about the test’s sensitivity and a second about its specificity. Having a hypothesis about sensitivity only is usually pointless for quantitative tests, since one can always arbitrarily set the test positivity rate, by changing the positivity threshold, to match the desired sensitivity. That, in itself, does not guarantee that the corresponding specificity is sufficiently high for the test to be clinically useful. The same applies to only having a hypothesis about specificity.

Informative tests produce a higher rate of positive test results in patients with the target condition than in those without the target condition. In ROC (receiver operating characteristic) space, the combination of sensitivity and specificity for these tests will then be in the upper left triangle (Fig. 2). Yet, in contrast to RCTs of interventions, where a null hypothesis of “no effect” works quite well in most cases, a null hypothesis of “not informative” is not very useful for evaluations of the clinical performance of diagnostic tests. Such a hypothesis may be relevant in the early discovery phase of biomarkers, but it will no longer be informative when a test has been developed, based on that marker, and when that test is evaluated for its added value to clinical practice. By the time a diagnostic accuracy study is initiated, one usually already knows that the test to be evaluated is more informative than just throwing a dice.

For many tests, both sensitivity and specificity will be higher than 0.50. A very simple study hypothesis then stipulates that both sensitivity and specificity be at least 0.50:

*H*_*1*_: {Sensitivity ≥ 0.50 *and* Specificity ≥ 0.50}

This could be evaluated against the following joint null hypothesis:

*H*_*0*_: {Sensitivity < 0.50 *and/or* Specificity < 0.50}

This hypothesis is also not very helpful in evaluations of the clinical performance of tests, because it can be too lenient in some cases and too strict in others. For example, if a test is meant to rule out disease, the number of false negatives should clearly be low. This means that a very high sensitivity is required, and a value barely exceeding 0.50 will not be enough. A useful triage test may combine a sensitivity of 0.999 with a specificity of 0.30, since it would mean that the triage test prevents further testing in 30% of those without the target condition, while missing only 1 in a 1000 in those with the target condition. If one wants a new, expensive test to replace an existing, inexpensive test, the accuracy of that new test should substantially exceed that of the existing test. Simply concluding that sensitivity and specificity exceed 0.50 will not be enough.

From these examples, we can conclude that the required levels of sensitivity and specificity will depend on the clinical context in which the new test will be used. This implies that we should explore that context explicitly when specifying hypotheses. Therefore, what would be more useful to know is not whether tests are informative, but whether they are informative enough, or in other words, whether the test meets “minimally acceptable criteria” (MAC) for a pre-defined test performance, i.e., levels of sensitivity and specificity that would justify the intended use. The study hypotheses then become:

*H*_*1*_: {Sensitivity ≥ MAC (Sensitivity) *and* Specificity ≥ MAC (Specificity)}

*H*_*0*_: {Sensitivity < MAC (Sensitivity) *and/or* Specificity < MAC (Specificity)}

In ROC space, this can be defined as a rectangle in the upper left corner that corresponds to MAC (Fig. 2). The test will be considered acceptable if both the sensitivity and specificity are in this rectangle, which we will refer to as the “target region” in ROC space.

A diagnostic accuracy study will produce point estimates of sensitivity and specificity, along with confidence intervals around it. If we position these in ROC space, then both the point estimates and the confidence intervals should be completely positioned in the target region. If MAC for sensitivity is set at 0.85 and MAC for specificity at 0.90, the lower limit of the confidence interval for sensitivity should exceed 0.85, and for specificity, it should exceed 0.90.

## Targeted test evaluation: defining minimally acceptable criteria for diagnostic accuracy

**Fig. 3:**
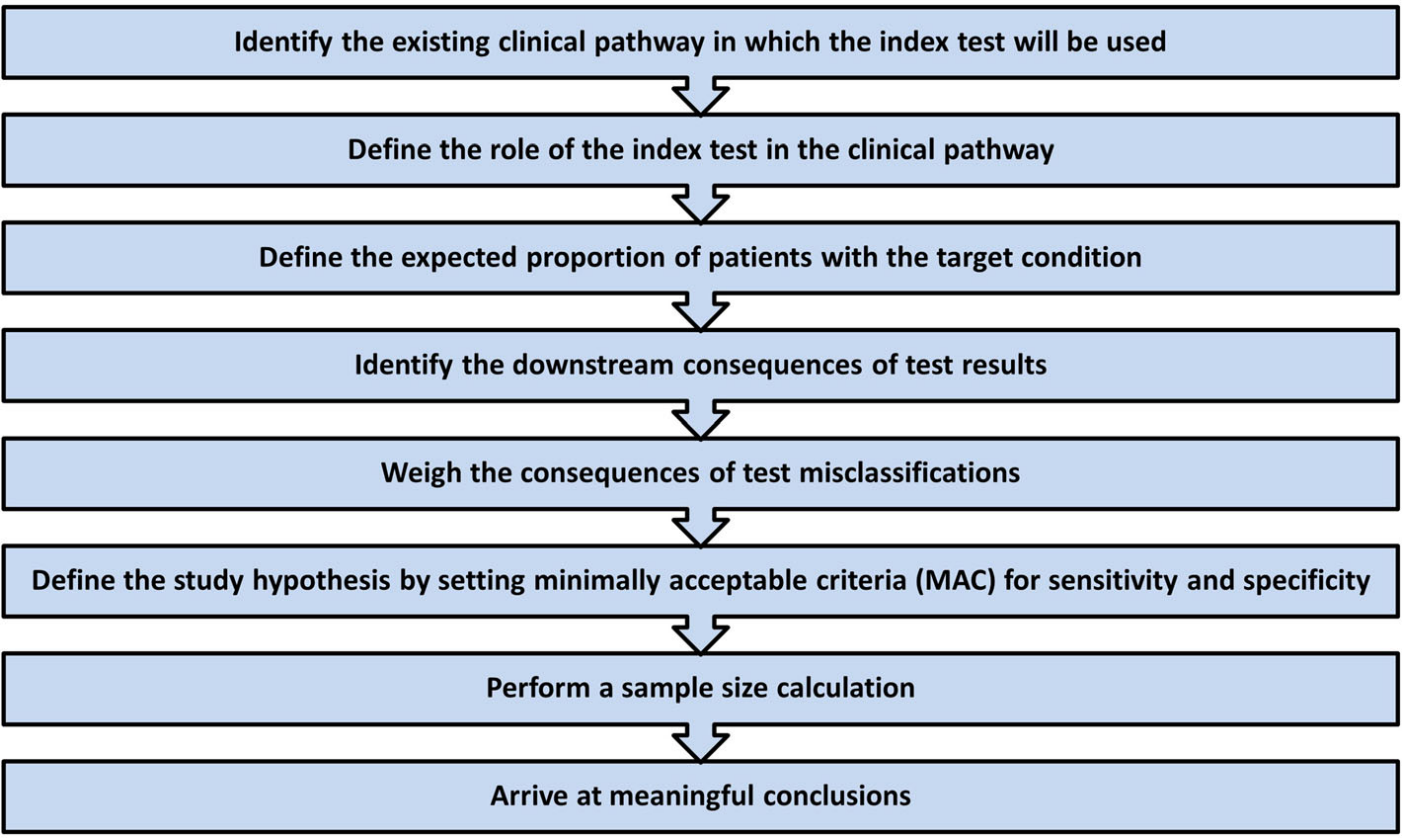
Defining minimally acceptable criteria (MAC) for diagnostic accuracy

**Fig. 4:**
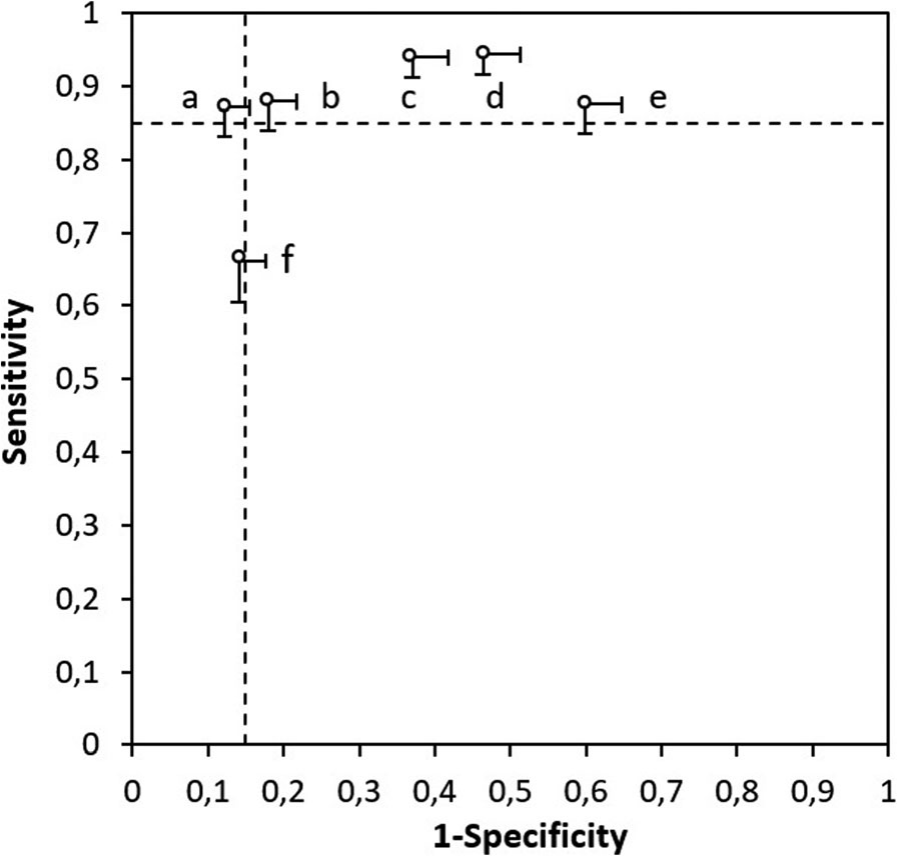
External validation of the diagnostic accuracy of rules-based selective testing strategies (figure derived from Cohen and colleagues [16]). Graph shows sensitivity and specificity estimates with their one-sided rectangular 95% confidence regions. Numbers indicate the rules-based selective testing strategies

Below, we provide a series of steps that could be used for defining minimally acceptable criteria for diagnostic accuracy (Fig. 3). A case example for each of the steps is reported in Table 3 and Fig. 4.

**Table 3:** Working example on how to define minimally acceptable criteria (MAC) for diagnostic accuracy

Identify the existing clinical pathway in which the index test will be used	
In children with pharyngitis, about one third of cases are due to bacterial infection with group A *Streptococcus* (GAS); the remainder are caused by viral infections [15]. Because of overlapping symptoms, the distinction between GAS and viral pharyngitis is clinically difficult. Cohen and colleagues aimed to externally validate existing clinical prediction rules that combine signs and symptoms for diagnosing GAS pharyngitis [16]. The existing clinical pathway is defined as follows: *• Target condition*. GAS pharyngitis. *• Targeted patients*. Children aged 3–14 years, with a diagnosis of pharyngitis, who have not yet received antibiotics. *• Setting*. Private office-based pediatricians. *• Tests in the existing clinical pathway*. Existing guidelines are not uniform on the clinical pathway for diagnosing and treating GAS pharyngitis. French guidelines recommend that all patients with pharyngitis undergo rapid antigen detection testing or throat culture to distinguish between GAS and viral pharyngitis [17]. North American guidelines, however, recommend that clinicians select patients for additional testing based on clinical and epidemiologic ground [18]. In clinical practice, children with pharyngitis are often treated with antibiotics without any additional testing [19].	
Define the role of the index test in the clinical pathway	
In case of a GAS pharyngitis, clinical guidelines recommend treatment with antibiotics. Misdiagnosis of GAS pharyngitis, however, could lead to unnecessary initiation of antibiotic treatment. Rapid antigen detection testing has a high specificity, but a sensitivity around 86%, which may lead to false-negative results [20]. Throat culture is considered the reference standard for GAS pharyngitis, but it may take up to 48 h before results are available, which causes delays in the initiation of treatment. The aim of clinical decision rules (the index test) is to identify patients at very low or very high risk, in whom additional testing can be safely avoided. In this setting, such a decision rule would serve as triage test.	
Define the expected proportion of patients with the target condition	
In establishing MAC for sensitivity and specificity, the authors assumed “a prevalence of group A streptococcal infection of 35%” [16], referring to a meta-analysis on the prevalence of GAS pharyngitis in children [15].	
Identify the downstream consequences of test results	
The aim of the study is to identify a clinical decision rule that is able to accurately detect patients at low risk or at high risk of GAS pharyngitis [16]. Patients at low risk will not receive antibiotics, as GAS pharyngitis is ruled out with a sufficiently high level of certainty; patients at high risk will receive antibiotics. No additional testing will be performed in either of these groups. This implies that patients falsely considered at high risk (i.e., false-positive results due to suboptimal specificity) will unnecessarily receive antibiotics with the inherent risk of adverse drug reactions, costs, and antibiotic resistance. Patients falsely considered as at low risk (i.e., false-negative results due to suboptimal sensitivity) will be withheld from adequate treatment with the risk of complications (e.g., retropharyngeal abscess, acute rheumatic fever, rheumatic heart disease), longer duration of symptoms, and risk of transmission of bacteria to others. Patients at intermediate risk based on the clinical prediction rule (neither at high risk nor at low risk for GAS pharyngitis) would still be selected to undergo additional testing (rapid antigen detection testing or throat culture), and a clinical prediction rule would not affect their clinical outcome.	
Weigh the consequences of test misclassifications	
In weighing the consequences of test misclassifications for sensitivity, the authors refer to expert opinion in previous literature: “Clinicians do not want to miss GAS cases that could transmit the bacterium to other individuals and/or lead to complications. […] Several clinical experts consider that diagnostic strategies for sore throat in children should be at least 80–90% sensitive” [16]. They weigh the consequences of test misclassifications for specificity as follows: “Assuming a population of a 100 children with pharyngitis and a GAS prevalence of 35%, a diagnostic strategy with 85% sensitivity would lead to 30 prescriptions for antibiotic therapy for 100 patients. We aim to identify a diagnostic strategy that could reduce the antibiotics consumption (baseline ≥60%). If we set the maximum acceptable antibiotics prescription rate to 40%, then the maximum acceptable number of antibiotics prescribed for GAS-negative patients would be 10 for 65 patients, for a specificity of 85%.”	
Define the study hypothesis by setting minimally acceptable criteria (MAC) for sensitivity and specificity	
The authors define MAC for sensitivity and specificity as follows: “After reviewing the literature and discussing until consensus within the review team, and assuming a prevalence of GAS infection of 35% and a maximally acceptable antibiotics prescription rate of 40%, we defined the target zone of accuracy as sensitivity and specificity greater than 85%. For each rules-based selective testing strategy, we used a graphical approach to test whether the one-sided rectangular 95% confidence region for sensitivity and specificity lay entirely within the target zone of accuracy” [16]. This means that the null hypothesis in this study can be defined as: *H*_*0*_: {Sensitivity < 0.85 and/or Specificity < 0.85}	
Perform a sample size calculation	
Since the aim of the study was to externally validate clinical prediction rules in an existing dataset, no sample size calculation was performed, which the authors acknowledge as a limitation in their discussion section: “A further limitation lies in the absence of an a priori sample size calculation. One of the clinical prediction rules met our target zone of accuracy based on the point estimates alone (Attia’s rule), but it was considered insufficient because the boundaries of the confidence intervals for sensitivity and specificity went across the prespecified limits for significance. This could be due to lack of power, and our results should be considered with caution until they are confirmed with a larger sample of patients” [16]. When using the calculator proposed in Additional file 1, the sample size calculation could have looked as follows. The MAC for sensitivity and specificity was set at 0.85; the authors provided no information on the expected sensitivity and specificity. This can, for example, be based on previous literature or on a pilot study. Assuming an expected sensitivity of 0.92 (with α* = 0.05, and β* = 0.90), 179 participants with the target condition (i.e., GAS infection) need to be included to ensure that the lower limit of the one-sided confidence interval for sensitivity is at least 0.85. Assuming an expected specificity of 0.95, 76 participants without the target condition (i.e., no GAS infection) need to be included to ensure that the lower limit of the one-sided confidence interval for specificity is at least 0.85. Taking into account an expected prevalence of GAS infection of 35% in the investigated population, this means that a total of at least 511 (= 179 × 0.35) participants with suspected GAS pharyngitis need to be included.	
Arrive at meaningful conclusions	
In their article, the authors graphically illustrate the performance of the investigated clinical prediction rules in ROC space (Fig. 4) [16]. The graphic shows that for five of the prediction rules, either sensitivity or specificity is outside the “target region”; for one prediction rule, both sensitivity and specificity are within the target zone, but the confidence intervals reach outside, which means that the null hypothesis cannot be rejected. Based on this, the authors conclude: “On external validation, none of the rules-based selective testing strategies showed sufficient accuracy, and none were able to identify patients at low or high risk whose condition could be managed without microbiologic testing.”

### Identify the existing clinical pathway in which the index test will be used

The diagnostic accuracy of a test is not a fixed property: it typically varies depending on the clinical setting in which it is applied, and on how the test is used [21]. Consequently, the sensitivity and specificity of a single test are likely to differ across settings and applications. Consequences of testing may also vary across different settings. Tests, therefore, should be evaluated in a setting that mirrors the clinical context in which they will be used. This can only be done by first defining the existing clinical pathway.

The identification of a clinical pathway is recommended in the evaluation of a diagnostic test by agencies such as the US Preventive Services Task Force (USPSTF); the Agency for Healthcare Research and Quality (AHRQ); the Grading of Recommendations, Assessment, Development and Evaluation (GRADE) Working Group; and the Cochrane Collaboration [22, 23]. Likewise, the STARD (Standards for Reporting Diagnostic Accuracy) 2015 statement recommends authors to report the intended use and clinical role of the index test [24, 25].

To help define the existing clinical pathway, we propose a number of guiding questions that authors of diagnostics accuracy tests can use:

- *What is the target condition to be diagnosed*? The target condition can be defined as the disease, disease stage, or severity or, more generally, the condition that the investigated test is intended to detect.
- *Who are the targeted patients?* The patients undergoing testing can be those presenting with certain signs or symptoms, or those having undergone specific previous tests, or just selected based on age, sex, or other risk factors, as in screening.
- *In which setting will the test be used?* The setting may be primary, secondary, or tertiary care, or, more specifically, the emergency department, outpatient clinic, or in the general community.
- *What are the other tests in the existing clinical pathway*? The test under evaluation may be positioned before or after other tests in the specific clinical setting as defined in the guiding question above. Also, a number of additional testing procedures may need to be considered, depending on the results of testing, before the diagnostic work-up can be closed and a clinical decision on further management is taken.

### Define the role of the index test in the clinical pathway

Defining the role of the index test in the existing clinical pathway is critical for defining eligibility criteria for participants for the study. This step involves defining where in the existing clinical pathway the test under evaluation will be positioned. There are several possible roles for diagnostic tests relative to an existing test—triage, add-on, replacement, or new test [26, 27]:

- A *triage* test is used before the existing test(s), and its results determine which patients will undergo the existing test.
- An *add-on* test is used after an existing test to improve the diagnostic accuracy of the testing strategy.
- A *replacement* test aims to replace an existing test, either because it is expected to have higher diagnostic accuracy, is less invasive, is less costly, or is easier to use than the existing test.
- A *new test* is a test that opens up a completely new test-treatment pathway. The latter would be the case with a new population screening strategy, for example, where, at present, no screening for the target condition is performed.

### Define the expected proportion of patients with the target condition

Depending on the proportion of tested patients who have the target condition, absolute numbers of false-positive and false-negative results will vary. If 100 patients are tested by a test with a sensitivity of 0.90 and a specificity of 0.90, and 50 of them have the target condition, one can expect, on average, 5 false positives and 5 false negatives. However, when only 10 of the 100 have the target condition, there will only be 1 false negative versus 9 false positives, even if these are tested with the very same test. As a consequence, the potentially harmful downstream consequences of the test will depend on how many of the tested patients have the target condition.

Several strategies can be used for defining the expected proportion of those with the target condition in a specific clinical setting. Ideally, a systematic review is identified or performed, to estimate this proportion, and to define relevant determinants. Alternatively, or additionally, a small pilot study can be performed, or clinical experts consulted.

### Identify the downstream consequences of test results

Bearing in mind the positioning of the index test in the clinical pathway, the downstream consequences of test results (i.e., test positives and test negatives) need to be defined. These refer to clinical management decisions, such as additional confirmatory tests patients may undergo if they are considered positive, or treatments that may be initiated or withheld as a result. Explicitly defining downstream consequences of the index test is important as they also determine the extent to which index test misclassifications (false-positive and false-negative results) could lead to harm to patients being tested.

### Weigh the consequences of test misclassifications

Defining MAC for sensitivity and specificity comes down to weighing the downstream consequences of test misclassifications: false-positive results versus false-negative results. Depending on what role the index test has in the clinical pathway, and the downstream consequences of being falsely positive or negative, this can influence the weight given to the consequences of being misclassified. Take for example, triage tests aimed at ruling out disease. These typically need to have high sensitivity, while specificity may be less important. In such a scenario, the consequence of being false negative may have the potential of being more detrimental than being false positive as one might not want to miss any potential true positive cases at the triage stage of a disease especially if early detection and treatment are crucial. Further down the clinical pathway, however, it may be crucial to keep the number of false positives to a minimum, since positive test results may lead to radical treatment decisions with potentially serious side effects. Therefore, add-on tests generally require higher specificity than triage tests. In other words, the weight given to the consequences of being false positive are higher in this scenario. For replacement tests, sensitivity and specificity should, commonly, be both at least as good as those of the existing test. When weighing the consequences of test misclassifications, the following should eventually be considered:

- Considering 100 patients suspected of the target condition, how many false-negative results are acceptable, considering the potential harms of such misclassifications?
- Considering 100 patients suspected of the target condition, how many false-positive results are acceptable, considering the potential harms of such misclassifications?

### Define the study hypothesis by setting minimally acceptable criteria for sensitivity and specificity

Based on the weighted consequences of false-positive and false-negative test results and taking into account the expected proportion of patients with the target condition (as defined earlier), MAC for sensitivity and specificity can be defined and the target region in the ROC space can be drawn (Fig. 2).

Pepe and colleagues recently provided a relatively simple method for specifying MAC that is based on weighing the harms and benefits of being detected with the target condition [28]. Their approach focuses on the threshold for starting the next action: the minimally required probability, after testing, of having the target condition that would justify subsequent management guided by testing, such as starting treatment, or order additional testing after a positive test result. From this threshold, and from the proportion of those with the target condition in the group in which the test under evaluation is going to be used, they derive minimum likelihood ratios: the combinations of sensitivity and specificity that would lead to the required post-test probability.

In their article, Pepe and colleagues argue that such thresholds can be inferred from comparisons with existing situations in which comparable actions are justified. An example is the probability of having colorectal cancers or its precursors in those referred for colonoscopy in a population screening program for colorectal cancer. A new marker would have MAC for sensitivity and specificity that would lead to a post-test probability that at least exceeds that probability.

The minimum positive likelihood ratio defines a specific region in ROC space: a triangle that includes the upper left corner. This area also includes very low values of sensitivity, which may not be clinically useful. The approach of Pepe and colleagues can be further refined by defining the acceptable number needed to test. This is the number of patients that must undergo testing in order to generate one positive result. It is the inverse of the positivity rate which depends on the proportion tested with the target condition and on the sensitivity and specificity. For expensive, invasive, or burdensome tests, the acceptable number needed to test will be lower than for simple, less costly tests.

Our framework focuses on weighing the consequences of test classifications for arriving at MAC for sensitivity and specificity. There are obviously other appropriate methods to define these. One option is to perform a survey among a panel of experts, directly asking what they would consider an appropriate MAC. Gieseker and colleagues, for example, evaluated the accuracy of multiple testing strategies for diagnosing *Streptococcus pyogenes* pharyngitis (“strep throat”); they performed a sample survey of pediatricians to identify a MAC for sensitivity and report: “67 (80%) of 84 were willing to miss no more than 5% of streptococcal infections” [29]. A similar method was used to identify minimally acceptable interpretative performance criteria for screening mammography [30]. In some areas, there are clearly established MAC. In triaging strategies to safely exclude pulmonary embolism without imaging, for example, it is now a common practice to require that the 3-month thrombo-embolic risk does not exceed 3% in test-negatives. This failure rate corresponds to that observed after a negative pulmonary angiography [31].

### Perform a sample size calculation

Based on the MAC for sensitivity and specificity and the expected proportion of patients with the target condition, a sample size calculation can be performed, which represents the number of participants (i.e., patients suspected of having the target condition) that need to be included in the study to conclude that the point estimates and lower limits of the confidence intervals for sensitivity and specificity fall within the “target region,” by rejecting the null hypothesis that they do not. The statistical tests and methods for sample size calculations have all been defined before in the literature [32].

Additional file 1 provides an example of a sample size calculator that can be used for this purpose, with background information on the formula used in Additional file 2. Information that needs to be filled in are α and β (see Table 1 for details), MAC for sensitivity and specificity, and the expected value for sensitivity and specificity. The output of the calculator is the minimal numbers of participants with and without the target condition that need to be included; the final sample size will depend on the expected prevalence of the target condition.

### Arrive at meaningful conclusions

Upon completion of the study, estimates of sensitivity and specificity are compared with the pre-defined MAC for sensitivity and specificity. This can be done by (1) assessing whether the point estimates of sensitivity and specificity and the lower confidence interval limits are above MAC, or (2) by performing formal statistical testing of the null hypothesis and arriving at a *p* value. As diagnostic accuracy studies have a joint hypothesis (one for sensitivity and one for specificity), one cannot reject the null hypothesis if only one of these fulfills the criteria for MAC and the other does not. One can also not reject the null hypothesis if the lower confidence limit of sensitivity or specificity is below MAC. Obviously, this “statistically negative” result does not mean that the diagnostic test is useless. Firstly, one should consider the possibility that the study was too small, for example, due to incorrect assumptions during the sample size calculations, which may have led to wide confidence intervals. Secondly, one should consider that the pre-specified criteria for MAC may have been too strict, or that the test may have added value in another clinical setting, or in a different role in the existing clinical pathway. On the other hand, a significant *p* value does not mean that the test under evaluation is fit-for-purpose; the study may be biased (e.g., due to many missing results) or have low generalizability.

## Conclusions

Targeted test evaluation will usually require the expertise of multiple professionals. There should be clinical experts to identify the management actions that will result from positive or negative test results and who can weigh the downstream consequences of test results. In some cases, it may be desirable to also include patients or their advocates in this process. There should also be methodological and statistical experts, to avoid mistakes in drawing the clinical pathway, to promote consistency in the process, and to arrive at adequate sample size calculations based on the defined MAC for test accuracy.

There is a growing recognition that explicitly specifying study hypotheses and how these were specified in the protocol-development phase of the study is crucial in test accuracy research. The STARD 2015 statement for reporting diagnostic accuracy studies now requires authors to report “study hypotheses” (item 4) and the “intended sample size and how it was determined” (item 18) [24, 25]. Similar methods for focusing on MAC of test performance are also increasingly being implemented among systematic reviews and clinical guidelines. The Cochrane Handbook for Diagnostic Test Accuracy Reviews, for example, now encourages authors to describe the clinical pathway in which the test under evaluation will be implemented, including prior tests, the role of the index test and alternative tests, if applicable [23]. A similar practice is advised by the recently established GRADE (Grading of Recommendations Assessment, Development and Evaluation) quality assessment criteria for diagnostic accuracy studies, which encourages guideline developers to focus on and weigh consequences of testing [33].

The process described here is not that different from hypothesis formulation and sample size calculations in RCTs. Even though most superiority RCTs generally have a simple null hypothesis (i.e., no effect), the calculation of the required sample size depends on the definition of a “minimum important difference”: the smallest difference in the primary outcome that the trial should be able to detect. The DELTA (Difference ELicitation in TriAls) group recently provided a systematic overview of methods for specifying the target difference in RCTs [34]. These methods are subdivided in those for specifying an important difference (e.g., by weighing resource costs and health outcomes to estimate the overall net benefit of the intervention), those for specifying a realistic difference (e.g., through a pilot study), or both (e.g., through opinion seeking among health professionals).

We realize that our framework has some potential shortcomings. We focused on MAC for the sensitivity and specificity of a new test, and null hypotheses based on these criteria, to be used in the evaluation of a single test with dichotomous test results. Defining MAC may be more difficult in other situations, although the general principles should be the same. In some cases, for example, diagnostic accuracy studies do not focus on a single test but compare two or more tests or testing strategies. Hayen and colleagues have described how one can use meaningful measures and statistics in such studies, such as the relative likelihood ratios [27]. In other situations, the index test does not produce a dichotomous test result, but a continuous one. This is, for example, often the case with laboratory tests. We believe that our framework could, with some adaptations, also be useful in those cases, as evaluating continuous tests generally comes down to finding a clinically relevant test threshold at which the test is useful for ruling in or ruling out the target condition. Currently, studies on continuous test very often select an optimal threshold for sensitivity and specificity based on, for example, Youden’s index. In many cases, this leads to a test threshold that is clinically not useful as both sensitivity and specificity are too low for decision-making. An alternative theory would to pre-define MAC for sensitivity and specificity, as outlined, and investigate whether there is a test threshold that is able to fulfill these criteria.

Mainly due to technological innovations, the field of diagnostic testing evolves quickly. Premature incorporation of new diagnostic tests into clinical practice may lead to unnecessary testing, waste of resources, and faulty clinical decision-making. Defining MAC before initiating new diagnostic accuracy studies should improve methodological study quality and help draw more meaningful evidence synthesis of such studies.

## Supplementary information

**Additional file 1.** An example of a sample size calculator.

**Additional file 2.** Formulas used for the calculator provided in Additional File 1.

## Data Availability

Not applicable.
