# Supplementary material for: Targeted test evaluation: a framework for designing diagnostic accuracy studies with clear study hypotheses
Source: Diagn Progn Res. 2019 Dec 19;3:22. doi: 10.1186/s41512-019-0069-2 (PMC6921417; doi:10.1186/s41512-019-0069-2)
Supplement: Supplementary file 2 — Additional file 2. Formulas used for the calculator provided in Additional File 1. [file 41512_2019_69_MOESM2_ESM.docx]

**Additional File 2**

Formulas used for the calculator provided in Additional File 1 are as follows.

The one-sided joint null hypothesis can be written as:

*H_0_ : {Sens ≤ Sens_0_ and/or Spec ≤ Spec_0_}*

The null hypothesis is tested by calculating a joint rectangular *1-α* confidence region for *{Sens, Spec}*.

The joint rectangular confidence region is made up of the cross-product of two one-sided,

$1-\alpha*=\sqrt{1-\alpha}$ confidence intervals.

For the required number of diseased (based on the minimally acceptable value of sensitivity):

$$D={\frac{(Z^{1-\alpha*}\sqrt{{Sens}_{0}\left( 1-{Sens}_{0} \right)}+Z^{1-\beta*}\sqrt{{Sens}_{1}\left( 1-{Sens}_{1} \right)})}{{({Sens}_{1}-{Sens}_{0})}^{2}}}^{2}$$

For the required number of non-diseased (based on the minimally acceptable value of specificity):

$$ND={\frac{(Z^{1-\alpha*}\sqrt{{Spec}_{0}\left( 1-{Spec}_{0} \right)}+Z^{1-\beta*}\sqrt{{Spec}_{1}\left( 1-{Spec}_{1} \right)})}{{({Spec}_{1}-{Spec}_{0})}^{2}}}^{2}$$

Where

$\alpha*=1-\sqrt{1-\alpha}$

$$\beta*=1-\sqrt{1-\beta}$$

$$Z^{1-\alpha*}=\phi^{-1}(1-\alpha*)$$

$$Z^{1-\beta*}=\phi^{-1}(1-\beta*)$$

*D* is the required number of diseased

*ND* is the required number of non-diseased

*Sens_0_* is the minimally acceptable value for sensitivity

*Spec_0_* is the minimally acceptable value for specificity

*Sens_1_* is the anticipated value of sensitivity

*Spec_1_* is the anticipated value of specificity

As usual,

$\alpha$ is the is the probability of Type I error in the joint hypothesis test
(incorrectly rejecting the null hypothesis *H_0_*).

$\beta$ is the probability of Type II error in the joint hypothesis test
(incorrectly failing to reject the null hypothesis; 1 – β is the power).

Notes:

- For sample size calculations, one has to set:

- Type I and type II errors for the joint hypothesis being tested (i.e., *α* and *β*)
- Minimally acceptable accuracy values (i.e., *Sens*_0_ and *Spec_0_*)
- Expected accuracy values (i.e., *Sens_1_* and *Spec_1_*)

- All calculations are made under the asymptotic normal approximation.

- All calculations are made under the assumption that sensitivity and specificity are calculated on independent samples.

More details can be found in:

Pepe M. S. (2003). The statistical evaluation of medical tests for classification and prediction. Oxford University Press, New York. Chapter 8: Study design and hypothesis testing, Section 8.2: Sample sizes for phase 2 studies

Available online at https://research.fhcrc.org/content/dam/stripe/diagnostic-biomarkers-statistical-center/files/excerpt.pdf
